# Formative Evaluation of an Early Family-Centred Prevention Programme for Childhood Overweight and Obesity (FruehstArt): A Study Protocol

**DOI:** 10.3390/children12121613

**Published:** 2025-11-26

**Authors:** Katharina Ruettger, Verena Fluegel, Anna Hagemeier, Kerstin D. Rosenberger, Martin Hellmich, Christine Joisten, Laura Mause, Nadine Scholten, Julia Glaubach, Miriam Hehn, Ida Bernhard, Marcus Redaèlli, Dusan Simic, Adrienne Alayli, Stephanie Stock, Kevin Dadaczynski

**Affiliations:** 1 Faculty of Human Science, Department Sports and Health Sciences, University of Potsdam, 14469 Potsdam, Germany; verena.fluegel@uni-potsdam.de (V.F.); kevin.dadaczynski@uni-potsdam.de (K.D.); 2 Institute of Medical Statistics and Computational Biology, Medical Faculty, University Hospital Cologne, University of Cologne, 50939 Cologne, Germany; anna.hagemeier@uni-koeln.de (A.H.); kerstin.rosenberger@uni-koeln.de (K.D.R.); martin.hellmich@uni-koeln.de (M.H.); 3 Department of Medical Statistics, University Medical Center Göttingen, 37073 Göttingen, Germany; 4 Department for Physical Activity in Public Health, Institute of Movement and Neurosciences, German Sport University Cologne, 50933 Cologne, Germany; c.joisten@dshs-koeln.de; 5 Institute of Medical Sociology, Health Services Research and Rehabilitation Science, Department of Health Services Research, Faculty of Medicine, University Hospital Cologne, University of Cologne, 50923 Cologne, Germany; laura.mause@uk-koeln.de (L.M.); nadine.scholten@uk-koeln.de (N.S.); 6 Centre for Health Communication and Health Services Research, Department for Psychosomatic Medicine and Psychotherapy, University Hospital Bonn, 53127 Bonn, Germany; 7 Institute of Health Economics and Clinical Epidemiology, Faculty of Medicine, University Hospital Cologne, University of Cologne, 50935 Cologne, Germany; julia.glaubach@uk-koeln.de (J.G.); miriam.hehn@uk-koeln.de (M.H.); ida.bernhard@uk-koeln.de (I.B.); marcus.redaelli@uk-koeln.de (M.R.); dusan.simic@uk-koeln.de (D.S.); adrienne.alayli@med.uni-duesseldorf.de (A.A.); stephanie.stock@uk-koeln.de (S.S.); 8 Department of General Pediatrics, Neonatology and Pediatric Cardiology, Medical Faculty, University Hospital Düsseldorf, Heinrich Heine University Düsseldorf, 40225 Düsseldorf, Germany; 9 Centre for Applied Health Science, Leuphana University Lueneburg, 21335 Luneburg, Germany

**Keywords:** childhood obesity, formative evaluation, prevention programme, mixed methods, intervention quality

## Abstract

**Background:** Early childhood obesity is an urgent public health challenge, with long-term health risks. The 12-month fruehstArt intervention aims to improve healthcare for 3 to 6-year-olds with overweight and obesity in Germany through a family-centred approach, including home-based counselling with coaches, paediatric consultations, and a supportive web application for both German- and Turkish-speaking families. This process evaluation will examine the barriers and enabling factors critical for successful implementation, identify necessary adaptations to the intervention, and assess its quality and acceptability among families, coaches, and paediatricians. **Methods:** This formative evaluation will use a mixed-methods approach including qualitative and quantitative data. Semi-structured interviews will be conducted with parents, coaches, and paediatricians at two different time points. Interviews will be analysed using qualitative content analysis. An Implementation Quality Index assessing the four dimensions of dosage, adherence, quality of delivery, and participant responsiveness will be developed, based on data collected from coaches carrying out the home-based counselling and participating parents of the intervention group. Linear mixed models for repeated measures will be used to analyse the relationship between data of the Implementation Quality Index and the primary and secondary outcomes of the effectiveness evaluation. **Conclusions:** The formative evaluation of the fruehstArt intervention is expected to identify important determinants of the implementation and provide valuable insights for future strategies to improve implementation. By identifying barriers and facilitators to participation, this study aims to pave the way for an effective dissemination of the intervention and ultimately contribute to improved health outcomes for children.

## 1. Introduction

Overweight and obesity are major global health challenges, affecting the physical, mental, and social well-being of millions of people [1,2]. In 2022, the World Health Organization (WHO) highlighted that worldwide more than 37 million children under the age of five were overweight [3]. Childhood obesity is associated with an increased risk of serious health problems in adult age such as cardiovascular diseases, diabetes mellitus, and mental disorders, which impact educational success and increase the risk of persistent obesity into adulthood [4,5,6,7,8,9]. Selective and indicated prevention strategies in early childhood are therefore crucial to mitigate its adverse short- and long-term effects and to promote healthier lives in adulthood [10].

The German Health Interview and Examination Survey for Children and Adolescents (KIGGS) found that approximately 14% of girls and 8% of boys in Germany aged 3 to 6 years are overweight or obese, with the prevalence varying by age, gender, and socioeconomic status (SES) [11]. Furthermore, the COVID-19 pandemic has exacerbated this problem, contributing to an increase in overweight and obesity in children and adolescents due to increased sedentary behaviour (e.g., screen time), reduced physical activity, and altered dietary habits [12].

The causes of childhood overweight and obesity are linked to genetic, psychological, social and environmental factors, as well as family and lifestyle habits such as eating patterns, physical activity and media consumption [13,14,15]. This underlines the importance of early intervention within the family setting, where habits are formed and can be most effectively influenced. Parents, as the primary architects of the family environment, play a key role in establishing these habits, highlighting the necessity of their involvement in interventions to prevent overweight and obesity [16,17,18]. By focusing on the family as a key setting for intervention, there is a great opportunity to address childhood overweight and obesity in a holistic and impactful way [19]. Paediatricians and other healthcare providers also play a crucial role in offering expert guidance to families and ensuring tailored, comprehensive approaches to prevention [20].

In Germany, the current care situation for children with overweight and obesity is characterised by insufficient and regionalised services with limited reach [21]. For the age group 3 to 6 years, there is no structured care in place, which represents a significant gap in research and intervention efforts [22]. Therefore, a family-centred, cross-sectoral care and prevention programme called fruehstArt, has been developed, specifically aiming at an early reduction of overweight and prevention of obesity in children. The fruehstArt programme is therefore conceptualised as a preventive approach that includes both overweight and obese children. While it does not represent primary prevention in the strict sense, its main objective is to prevent further weight gain among overweight children and to avoid the persistence of obesity into adolescence and adulthood among those who are already obese. Thus, fruehstArt combines elements of secondary and tertiary prevention, aiming to reduce long-term health risks through early, family-based lifestyle and behavioural interventions.

The aim of this part of this study is to conduct a formative evaluation of fruehstArt at the process level to analyse context-specific barriers and enabling factors that are critical for the implementation of the intervention, and to identify adaptions to the intervention’s structure as well as possible modifications in the implementation concept. It seeks to enhance understanding of the intervention’s implementation quality and factors influencing its acceptability among families, coaches, and paediatricians.

## 2. Methods

### 2.1. The FruehstArt Intervention

FruehstArt offers an innovative intervention for families with children aged 3 to 6 years who are overweight (Body Mass Index (BMI) > P (Percentile) 90) or obese (BMI > P97) or a sudden weight gain with a BMI-SDS (BMI Standard Deviation Score) increase of 0.2/year is detected, which is classified as a cause for concern by the paediatrician [23].

Given that empirical studies have found a higher prevalence of overweight and obesity among children from families with a migration background [24], fruehstArt addresses not only German-speaking but also Turkish-speaking families, which is the group with the highest percentage of migrants in Germany [25]. To support access to this population group, fruehstArt engages Cologne neighbourhood mothers—trained women with relevant cultural backgrounds—to facilitate community access and support coaches in their endeavour.

To achieve fruehstArt’s primary aim of reducing childhood overweight and obesity, it promotes physical activity, a balanced diet, healthy sleep patterns and an age-appropriate use of media. To achieve this aim, families participate in home-based coaching sessions conducted by trained coaches, who offer tailored counselling within the family context to meet the distinct needs of each family. These coaches cooperate with each child’s paediatrician to develop a customised counselling plan. Moreover, aligned with the socio-ecological approach of overweight and obesity, the coaches help families to identify and make use of regional health services [26,27]. These include e.g., sports clubs close to their homes, addressing environmental and community-level factors that support healthy behaviours. This introduces an innovative model of cross-sectoral support, merging behavioural and community-based support measures, thereby representing a comprehensive and tailored approach to healthcare for families.

**Figure 1:**
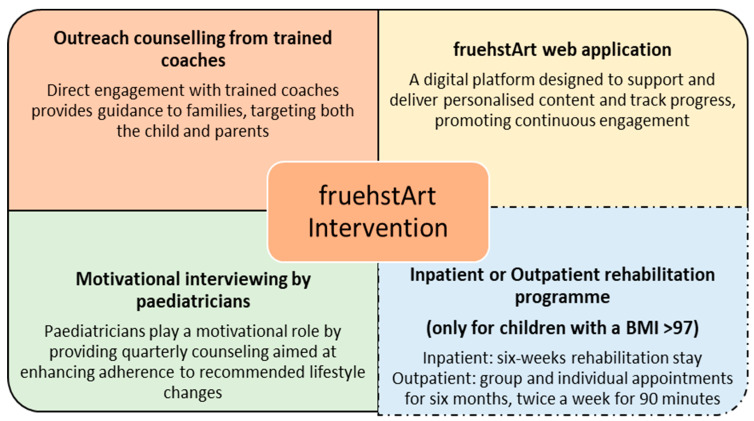
Key components of the fruehstArt intervention. Dashed line shows optional rehabilitation services for families.

Children are assigned to one of six coaching strands depending on the child’s BMI percentile and individual support needs [28]. Based on the individual needs of the family the number of coaching sessions varies, ranging from 10 to 20 home visits within the 12-month intervention period. Additionally, those with a BMI above the 97th percentile are offered a six-week inpatient rehabilitation service or a newly developed outpatient rehabilitation service, available twice a week for six months near their home. However, the rehabilitation services are optional for the families and are not an integral part of the intervention. At enrolment and during quarterly follow-up visits, all participating families receive counselling sessions conducted by paediatricians trained in Motivational Interviewing (MI), a client-centred counselling approach aimed at enhancing intrinsic motivation for lifestyle behaviour change. Prior to implementation, paediatricians attend a single 3.5 h training session (online or in person) or provide proof of equivalent prior training. The training is participatory in nature and includes role plays addressing typical counselling situations and challenges in daily practice. All families have access to the fruehstArt web application, where they can communicate with the coach, access a goal-setting tool or recipes, and participate in all study questionnaires. The key components of the intervention are shown in Figure 1.

### 2.2. The Evaluation Design of FruehstArt

The evaluation concept of this study employs a mixed-methods approach, incorporating a summative evaluation, formative evaluations at process and system level, and a health economic analysis. The project is funded by the Joint Federal Committee for a period of 48 months, from 2022 to 2026. The intervention is currently being piloted in North-Rhine-Westphalia, the most populous federal state of Germany.

The summative evaluation is conducted as a randomised controlled trial (RCT) including 812 children. It assesses the effectiveness of fruehstArt by analysing changes in the primary outcome, BMI-SDS, at 12 months in the intervention group (IG) in comparison to the control group (CG). Children are recruited from paediatric practices and obesity centres participating in the project and randomised in a 2:1 (541:271) ratio. Measurements of weight and height are conducted quarterly in paediatric practices.

The IG receives all key components of fruehstArt for 12 months, while families in the CG receive a motivational interview conducted by a paediatrician every four months and the current standard of care. The CG also has limited access to the fruehstArt web application for completing study questionnaires.

Parents will complete questionnaires at three time points: baseline (T0), six months (T1), and 12 months (T2) after enrolment. These questionnaires will be used to collect comprehensive data on a variety of characteristics, including parental health literacy, the child’s daily lifestyle habits, the parents’ level of physical activity and the parents’ quality of life. In addition, a motor skills test (side-to-side jumping), conducted by coaches and study coordinators at baseline (T0) and after 12 months (T2), is included as part of the summative evaluation to assess potential changes in children’s physical competence in relation to physical activity and weight development [29].

A more detailed overview of the overall evaluation design of fruehstArt can be found in the study protocol of the overall study [28]. The methodology relevant to this formative evaluation will be described below.

### 2.3. Objectives and Research Questions

The formative evaluation at the process level aims to identify barriers and facilitators of fruehstArt, the quality of the intervention, and the feasibility of implementation strategies. It also aims to describe and understand the contextual factors of the intervention to provide recommendations for adapting fruehstArt.

Based on the research objectives, the research questions that are addressed are as follows:

1. What factors facilitate or hinder the implementation of the intervention components?
2. What is the quality of implementation of fruehstArt and what differences can be observed between the families?
3. Does implementation quality influence the effectiveness of the fruehstArt intervention?

### 2.4. Study Design

**Figure 2:**
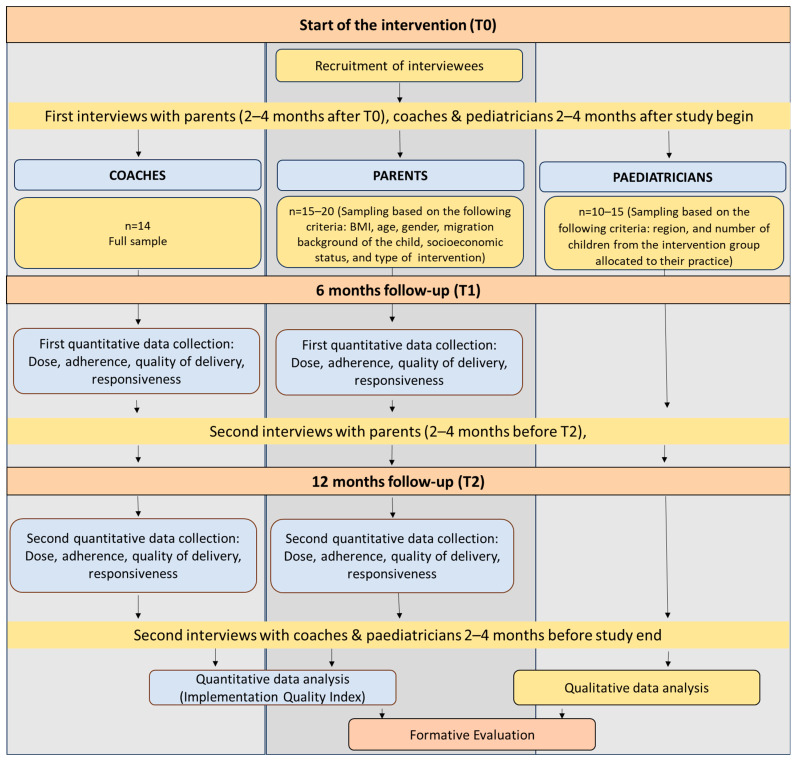
Overview of the study design for the formative evaluation at process level.

The formative evaluation strategy is based on a mixed-methods approach, incorporating both qualitative and quantitative data. This involves conducting interviews with participating parents, coaches, and paediatricians at two time points to gain a longitudinal understanding of their experiences and perspectives. Quantitative data will be collected to develop an index for measuring implementation quality. Both methods will be used following the recommendations of the UK Medical Research Council [30]. A schematic overview of the design of the formative evaluation at the process level is provided in Figure 2.

### 2.5. Participants and Recruitment Strategies

Participant recruitment for this study began in December 2023 and was completed in March 2024. Data collection is ongoing and is expected to continue until April 2026. The final data export is scheduled for June 2026, after which data analysis will be conducted.

Interviews

**Families** (*n* = 15 to 20) will be considered for an interview based on the assumption that this number is sufficient to reach theoretical saturation in qualitative research [31]. Families allocated to the intervention group will be selected based on the following criteria: BMI-SDS, age, gender, migration background, SES, and coaching strand. To ensure a heterogeneous sample, attention will be paid to distribution balance across these criteria. Families will be recruited every three months to include participants from different stages of study progression. The initial interview will be conducted between two and four months after the start of the intervention. A follow-up interview will be conducted between two and four months before the intervention ends.

**Paediatricians** (*n* = 10 to 15) will be considered for their first interview based on the assumption that this number is sufficient to obtain meaningful insights while ensuring theoretical saturation. They will be invited for an interview if at least four children have been assigned to their practice, ensuring they have acquired sufficient experience with the project’s processes to provide valuable reflections. The second interview is planned to take place two to four months before the end of fruehstArt. Paediatricians will be selected based on the following criteria: region and the number of children from the intervention group assigned to their practice. The number of interviews with paediatricians is lower, as the total number of participating paediatricians is considerably smaller than that of the families. However, this number remains flexible, and additional paediatricians will be recruited if necessary, e.g., if saturation is not reached.

**Coaches** (*n* = 14 in total) will be eligible for their interview once they have coached four families and delivered at least four coaching sessions per family. To identify changes that occur over the course of the project the second interview will be conducted two to four months before the end of fruehstArt.

Potential interviewees will be contacted via email or postal letter and provided with study details. They can choose between in-person, online, or telephone interviews, which will last approximately one hour, be conducted by one researcher, and audio recorded with written consent.

Implementation Quality Index

Quantitative data to measure the Implementation Quality Index will be collected at T1 and T2 from both the coaches (n = 14) and all parents (n = 541) in the intervention group. No separate sample size estimation was conducted for the formative evaluation, as it builds on the intervention group of the summative evaluation, for which a sample size calculation was performed. Data will be used from the fruehstArt app that includes a questionnaire specifically tailored and developed for the purpose of the formative evaluation study.

### 2.6. Instruments

Interviews

Interview guides were developed based on the Consolidated Framework for Implementation Research (CFIR) and consist of open-ended questions and probes to elaborate on answers for clarification [32,33]. The CFIR provides a comprehensive approach to understanding implementation factors across various settings and has been widely used in numerous formative evaluation studies to successfully identify context-specific barriers and facilitators during the implementation process, including mental health programmes [34] or obesity prevention programmes [35,36]. The CFIR comprises five major domains: (1) intervention characteristics, (2) inner setting, (3) outer setting, (4) characteristics of individuals involved, and (5) implementation process. These five domains are further categorised into 39 constructs and sub-constructs presenting implementation factors. A full description of the framework, including relevant interview questions and complete interview guides, can be found in Supplementary Materials Table S1. A brief description of the framework domains, including exemplary questions, is presented in Table 1. Prior to data collection, the interview guides were pilot-tested with the study coordinators, who are familiar with the context and all three target groups. Their feedback informed minor revisions to improve clarity, relevance, and flow of questions. This iterative process ensured face validity of the interview instruments and their suitability for the intended purpose. To reduce potential social desirability bias, participation was voluntary and respondents were assured of confidentiality and anonymity. Interviews were conducted by trained researchers not involved in the intervention delivery, who emphasised the importance of honest and authentic responses.

Implementation Quality Index

As various research studies have demonstrated, implementation quality is a crucial factor in successful implementation of an intervention [37]. To capture this, we will develop an Implementation Quality Index for each participating family based on Dowling and Barry’s framework [38]. This index will assess the following dimensions of the intervention:

1. **Dosage**: The quantity of intervention modules used, such as the number and duration of coaching sessions, rehabilitation services accessed, and the utilisation of the fruehstArt application.
2. **Adherence**: The extent to which the intervention was delivered as intended (fidelity), including adherence to coaching guidelines and any deviations.
3. **Quality of Delivery**: The assessment of how well the intervention was delivered, as reflected in parents’ evaluations of their relationship with coaches and the coaching sessions themselves.
4. **Responsiveness**: Participants’ engagement with the intervention, including parents’ feedback on the fruehstArt web application and coaches’ evaluations of parental involvement.

These dimensions were specifically operationalised for fruehstArt. To establish face validity, the Implementation Index questions were pre-tested with parents not enrolled in this study to ensure that all items were clearly understood. Feedback from this pilot testing informed minor revisions to improve wording and clarity. Table 1 provides a detailed breakdown of the indicators.

### 2.7. Data Analyses

Interviews (Research Question 1)

Qualitative data will be analysed using qualitative content analysis, following the six steps outlined in Kuckartz’s method [39]. In the first step, audio recordings will be anonymised, transcribed verbatim by a member of the research team, and prepared for data analysis. Secondly, the main categories corresponding to the interview questions will be formed. In step three, codes will be generated within these main categories. In the next step, text passages of the main categories will be formed into subcategories. In step five, a category-based analysis will be conducted and reported in the final step. Throughout this process, a member of the research team will act as a critical friend, and challenge the author’s beliefs and offer feedback. Subsequently, themes will be deductively coded into the CFIR by the authors and again discussed with the critical friend. MAXQDA24 will be used for qualitative data analysis. Immediately after each interview, a team of researchers will deliberate on significant issues raised in the interview such as technical issues or problems with the workflow, and subsequently report these in writing to the study coordinator, where they are evaluated and discussed. Whenever feasible and necessary, adjustments are implemented promptly to enhance the overall quality of the intervention.

If participants decide not to take part in the second interview, their first interview will remain part of the analysis, unless they explicitly request deletion of their data, in which case all related material will be removed in accordance with ethical guidelines. All cases of attrition between the two interview rounds will be documented, and where possible, reasons for non-participation will be recorded. Thematic findings from both time points will be compared, with missing follow-up data considered in the interpretation of results.

To ensure culturally sensitive interpretation, the analysis will be conducted with cultural sensitivity, considering potential differences between German- and Turkish-speaking participants and discussing cultural nuances within the multilingual research team. Turkish-speaking coaches and neighbourhood mothers will contribute contextual insights to identify possible participation barriers and inform further adaptation of the intervention.

Implementation Quality Index (Research Question 2)

For the quantitative data analysis, all indicator values are aggregated at the family level and converted into a percentage value following the procedure outlined by Dowling and Barry [38]. This allows the individual indicator values within a dimension to be merged into an overall value between 0 and 1 for the respective dimension. The four overall ratings of the dimensions (dosage, adherence, quality of delivery, and responsiveness) are subsequently combined to yield a mean value for the overall quality of implementation.

$$\begin{array}{c} Total\;Implementation\;Quality\\ =\frac{\left(Total\;Dosage+Total\;Adherence+Total\;Quality\;of\;Delivery+Total\;Participant\;Responsiveness\right)}{4} \end{array}$$

The items and indicators are not weighted, ensuring that all dimensions contribute equally to the quality of implementation. The visual binning method [40] is utilised to divide the overall assessment into four distinct categories, which are defined by the mean value and ±1 standard deviation: low implementation quality, moderately low implementation quality, moderate high implementation quality, and high implementation quality. Consequently, the families are allocated to the corresponding level of implementation quality. 

Missing data will be handled using an Available Case Analysis (ACA) approach, assuming data are Missing Completely at Random (MCAR). In line with Dowling and Barry [38], cases with more than 25% missing data in key variables will be excluded from the analysis to avoid bias and maintain the reliability of results.

Impact of Implementation Quality on fruehstArt effectiveness (Research Question 3)

Mixed models for repeated measures will be used to analyse the relationship between the Implementation Quality and the primary (BMI-SDS) and secondary outcomes (see [28]) of the effectiveness analysis (summative evaluation).

**Figure 3:**
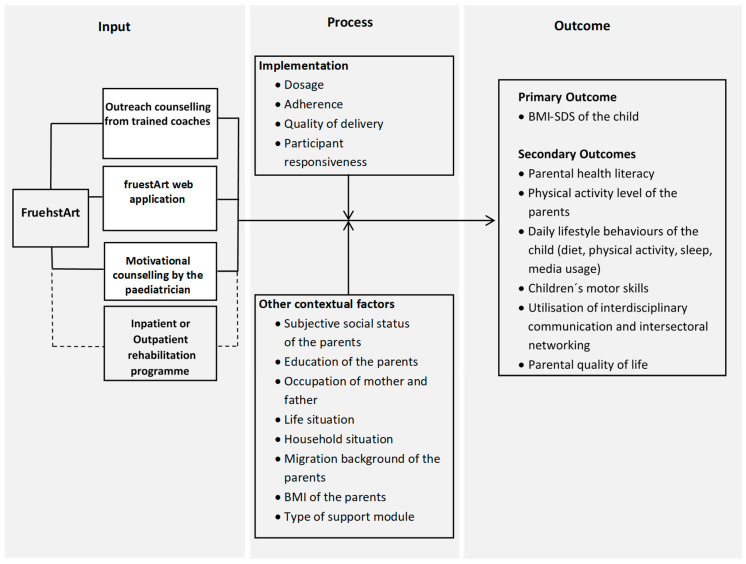
fruehstArt intervention model.

The intervention model shown in Figure 3 illustrates the relationships to be analysed and is divided into three main areas: Input, Process, and Outcomes. The ‘Input’ includes the previously described key components of the intervention, designed to work synergistically to support participants. The influence of implementation quality and other contextual factors will be analysed at the ‘process’ level as part of the formative evaluation. Certain variables within the implementation may act as mediators between the inputs and the outcomes (primary and secondary). The intervention model aims to capture the complex interactions between various implementation and contextual variables and their effects on the primary and secondary and serves as basis for modelling further statistical analysis.

## 3. Discussion

The results of the formative evaluation are expected to sustainably improve the quality of future implementation of fruehstArt through adapting the intervention’s structure and modifying the implementation concept. This will bridge the gap between current evidence and practical application in healthcare as there is a disconnect between what research shows to be effective and what is actually implemented in clinical and organisational settings [41].

By uncovering the factors that contribute to implementation quality and examining the relationship between implementation quality and intervention outcomes, this study will enhance our understanding of the key determinants of successful intervention implementation based on multi-level data collection from service providers and participating families. It will also elucidate how these determinants are associated with the effectiveness of the intervention.

Strengths

One of the strengths of this formative evaluation at process level is its mixed-methods design, which includes both qualitative and quantitative data from participating families and service providers (coaches and paediatricians). Evaluating an intervention from multiple perspectives allows a comprehensive understanding of the needs and preferences of families with overweight or obese children, as well as those of service providers. This helps to investigate whether the intervention was implemented accordingly to those needs and wishes of the target groups to increase its appeal and applicability. In addition, the provision of ongoing, interactive feedback facilitates immediate modifications to the intervention to ensure that it remains effective and relevant to participants’ needs. This study will also identify factors that may influence the feasibility of fruehstArt, such as availability of resources, time constraints of families or cultural and personal preferences regarding lifestyle choices.

The development of an Implementation Quality Index based on existing evidence and experience is another strength of this study, as it provides a valuable basis for linking quantitative data on implementation quality with the primary and secondary outcomes of the effectiveness analysis (summative evaluation). Linking the data will allow exploration of the conditions under which the fruehstArt intervention achieves its intended effects. By linking the data, it will be possible to determine not only whether the fruehstArt intervention is effective, but also under what conditions of implementation it is effective. This area has been underexplored in previous research, so the index facilitates systematic comparisons and increases the robustness of study conclusions, thus filling a significant gap in the existing literature.

Findings and Implications

Findings from the formative evaluation will inform the adaptation and further development of the fruehstArt intervention before potential large-scale implementation. Insights will be shared with consortium partners and relevant stakeholders to enable refinement of the programme and to derive implementation strategies, and policy recommendations for the prevention of childhood overweight and obesity in Germany.

Limitations

Evaluating processes in real time may miss longer-term challenges or successes that emerge after the evaluation period which can lead to bias and inaccuracy. Difficulties in recruiting participants may lead to a smaller sample size than anticipated, which could limit the validity of the results. Additionally, interviewing only a relatively small number of participating families and paediatricians may not adequately capture the diversity of experiences, perspectives, or contextual factors present across the full study sample and all service providers. Another major challenge is the management of missing data, which could introduce bias or reduce the statistical power of the analyses. However, missing data is being minimised through user-friendly data collection via an e-health platform and a regular reminder management.

Qualitative data also may introduce bias due to subjective interpretations by participants or researchers making it difficult to draw general conclusions about this study’s overall implementation process. The potential for selection bias also exists, as the families and paediatricians who participate might have a greater interest in the topic or better resources. Therefore, participating families and paediatricians might differ systematically from those who do not, potentially skewing the results.

Lastly, all indicators of the Implementation Quality Index were self-developed and therefore not psychometrically tested. This may limit the validity and reliability of the findings. To compensate for that, the development has undergone expert consultations also including communication with the authors who developed the original version of the index. Moreover, Adherence and Quality of Delivery were partly based on coach self-assessments, which may introduce bias. However, the inclusion of parental evaluations of coaching quality provides an additional external perspective, allowing us to validate and contextualise the coaches’ ratings.

In addition, all four dimensions of the Implementation Quality Index were weighted equally. While this approach follows the procedure proposed by Dowling and Barry [38] and allows for comparability, it may not fully capture potential differences in the relative importance of each dimension for implementation success. For this reason, the evidence base will be continuously monitored throughout the project so that adjustments can be made to the weighting of individual dimensions if necessary.

Conclusions and outlook

While this formative evaluation provides valuable insights into the implementation of fruehstArt, some limitations concerning the feasibility and generalisability of findings should be acknowledged. As implementation processes are highly context-dependent, the transferability of findings to other healthcare settings or populations should be interpreted with caution.

Moreover, the anticipated results have the potential to advance theoretical understanding of how contextual and behavioural factors influence implementation success in childhood obesity prevention. By applying and potentially refining constructs of the Consolidated Framework for Implementation Research (CFIR), this study may contribute to the ongoing development of theory-driven approaches to designing and evaluating preventive interventions in early childhood.

Finally, the results will also contribute to a stronger consideration of the connection between formative and summative evaluation. If, as assumed, a connection between implementation quality and effectiveness can be demonstrated, this would underpin the importance of implementation in the context of evidence-based intervention development and delivery.

If fruehstArt demonstrate effectiveness, it could serve as a model for broader implementation within the German healthcare system. A scaled-up version might support improvements in early prevention and intervention structures, thereby contributing to healthier developmental trajectories in early childhood.

## Figures and Tables

**Table 1:** Overview of quantitative and qualitative indicators.

	Main Research Question	Implementation Outcome	Definition	Participants	Example Questions	Time of Evaluation
**Implementation Quality Index** *(data source: fruehstArt app and questionnaire data)*	***What is the implementation quality of fruehstArt?***	*Dosage*	*Quantity of the intervention or intervention modules implemented and used*	*N = 541 Parents* *N = 14 Coaches*	*How often do you use the fruehstArt app (Parents)?* Reply options: 1 = less than once a month to 7 = multiple times per day *How long did the home visit last? (Coaches)* *Reply option:* *Duration in minutes*	*T1 and T2* *(6-month and 12-month follow-up)*
		*Adherence*	*The extent to which the intervention or intervention modules were implemented as intended*	*N = 14 Coaches*	The coaching sessions have been implemented as planned. *(Coaches)* Reply options: 1 = Does not apply to 4 = Fully applies	
		*Quality of delivery*	*Refers to how well the service providers implement an intervention or intervention modules*	*N = 541 Parents*	*How would you describe the quality of your collaboration with your coach? (Parents)* Reply options: 5 = Really good to 1 = Really bad	
		*Participant responsiveness*	*Subsumes the participants’ reactions to, and handling of the intervention*	*N = 541 Parents* *N = 14 Coaches*	*I have been able to put the content of the previous coaching sessions into practice (Parents)* Reply options: 1 = Strongly disagree To 4 = Strongly agree *How well did the family respond to the counselling methods used? (Coaches)* Reply options: 1 = Really responsive to 5 = not at all responsive	
**Qualitative Interviews** (open questions)	***What factors facilitate or hinder the implementation of the intervention components?***	*Intervention characteristics*	*The characteristics of the intervention itself, such as its strength of evidence, adaptability, complexity, costs*	*N = 14 Coaches* *N = 15–20 Parents* *N = 10–15 Paediatricians*	*How do you perceive the general working conditions of the coaching sessions (workload, journey,* preparation time? *(Coaches)* *What do you think about the intensity of fruehstArt* (e.g., *number of visits to the paediatrician, number of coaching sessions)? (Parents)* *In your opinion, what changes or adjustments would need to be made to the project so that it can be effectively embedded in the care of children with overweight and obesity in the long term? (Paediatricians)*	*Parents: 2–4 months after T0 and 2–4 months before T2* *Coaches and Paediatricians:* *2–4 months after study begin and 2–4 months before the study end*
		*Inner setting*	*The structure, culture, climate, and resources of the organisation that can influence implementation, such as leadership, communication, learning environment, willingness to change*	*N = 14 Coaches* *N = 15–20 Parents* *N = 10–15 Paediatricians*	*How do you experience the general communication in the fruehstArt project? (Coaches)* *How do you rate the supporting information provided in the fruehstArt app? (Parents)* *How willing is your staff to implement fruehstArt as planned?* *- Why is this? Can you explain? (Paediatricians)*	
		*Outer setting*	*The influences outside the organisation that can promote or hinder implementation, such as political, economic, social, or regulatory factors*	*N = 14 Coaches* *N = 15–20 Parents* *N = 10–15 Paediatricians*	*What obstacles do families face regarding the coaching sessions* (e.g., *time, communication, financial, cultural, parents’ level of education)? (Coaches)* *Was there anything that prevented you from attending the appointments with the paediatrician* (e.g., *work, illness, stress)? (Parents)* *To what extent did you feel external influences or pressures* (e.g., *politics, measures, COVID-19) that may have influenced your decision to participate at fruehstArt* (e.g., *rising obesity rates in children in your practice)? (Paediatricians)*	
		*Characteristics of individuals involved*	*The characteristics of the people involved in or affected by the implementation, such as their knowledge, attitudes, motivations, self-efficacy*	*N = 14 Coaches* *N = 15–20 Parents* *N = 10–15 Paediatricians*	*To what extent has the motivation and commitment of the families changed throughout the project? (Coaches)* *How confident do you feel about implementing the contents of fruehstArt even after the project has ended and setting yourself new goals? (Parents)* *Has your participation in fruehstArt changed the way you communicate and interact with your patients/families? (Paediatricians)*	
		*Implementation process*	*The activities and strategies used to plan, implement and evaluate implementation, such as stakeholder engagement, customisation of the intervention, monitoring of progress*	*N = 14 Coaches* *N = 15–20 Parents* *N = 10–15 Paediatricians*	*To what extent have you been able to carry out the home visits as planned? (Coaches)* *How did you perceive the first weeks of coaching? (Parents)* *Please describe how fruehstArt was introduced in your practice? Who was involved in the planning process? (Paediatricians)*	

## Data Availability

The data set will be available to all principal investigators. The datasets generated for the current study will not be publicly available but will be available from the corresponding author on reasonable request.

## Supplementary Materials

The following supporting information can be downloaded at: https://www.mdpi.com/article/10.3390/children12121613/s1, Table S1: CFIR domains including example questions.

## Abbreviations

The following abbreviations are used in this manuscript:

BDSG	German Federal Data Protection Act
BMI-SDS	BMI Standard Deviation Score
CFIR	Consolidated Framework for Implementation Research
CG	Control group
EU GDPR	European General Data Protection Regulation
IG	Intervention group
KIGGS	German Health Interview and Examination Survey for Children and Adolescents
MI	Motivational Interviewing
RCT	Randomised controlled trial
SES	socioeconomic status
T0	Baseline
T1	Six months
T2	12 months
WHO	World Health Organization
