# Supplementary material for: Formative Evaluation of an Early Family-Centred Prevention Programme for Childhood Overweight and Obesity (FruehstArt): A Study Protocol
Source: Children (Basel). 2025 Nov 26;12(12):1613. doi: 10.3390/children12121613 (PMC12732125; doi:10.3390/children12121613)
Supplement: Supplementary file 1 [file children-12-01613-s001.zip › children-3942282-supplementary.pdf]

Table S1: CFIR domains including example questions

	Constructs	Coaches	Paediatricians	Parents
**1. Intervention Characteristics**	**Evidence Strength and Quality** *(Stakeholders' perception of the quality and validity of the evidence that supports the assumption that the intervention will achieve the desired outcomes)*	Why did you decide to participate in the fruehstArt project as a coach?<br>- What motivated you? Were there any aspects that made you doubt your involvement in the project? (T1)	Why did you decide to participate in the fruehstArt project?<br>- What were your expectations? Did you have any concerns? (T1)<br><br>Are you aware of any information or evidence that proves the effectiveness of fruehstArt (e.g. information from published literature, colleagues, professional organisations, other sources, guidelines)?<br>If so: What do you think of the evidence? (T1)	
	**Relative Advantage** *(Stakeholders recognise the advantage of implementing the intervention over an alternative solution)*	Have you been involved in a prevention programme like fruehstArt in the past?<br>- If so, which one and in what role? (T1)<br><br>How do you rate fruehstArt in comparison to existing programmes/care? - Advantages, disadvantages (T1)<br><br>How did you perceive the range of rehab programmes	Have you previously participated in a similar programme with your practice in the past?<br>- If so, which one? (T1)<br><br>Please compare the fruehstArt project with the current healthcare situation.<br>- What are the differences? Advantages? Disadvantages? (T1)<br><br>How did you perceive the rehab programme (outpatient and inpatient) for the families?	Has your child or family already made use of or received support or counselling with regard to existing obesity in the past? What was your experience with it?<br>- Care in the paediatric practice? Socio-paediatric centre? Inpatient rehab? Sports clubs? Other clubs? (T1) What additional support would you have wished for when you learned about your child's overweight problem? (T1)<br><br>If you compare fruehstArt with other offers that you have used

		(outpatient & inpatient) for the families?<br>- Did the rehabilitation programme support the coaching process? In your opinion, did the rehab programme provide families with an additional advantage in their process? Or did it create additional barriers? (T2)<br><br>What differences have you noticed between the outpatient and inpatient rehab programme? (T2)	- Please explain. What differences did you notice between the outpatient and inpatient rehab programmes? (T2)	so far: In your opinion, does fruehstArt have any additional benefits? (T2)
	**Adaptability**<br>*(The extent to which an intervention can be adapted, tailored, refined or reinvented to meet local needs)*	How do you perceive the general conditions of the coaching sessions? (Workload, journey, hire car, preparation, follow-up, communication)<br>- What adjustments need to be done to the general conditions? (T1 & T2)<br><br>In your opinion, what changes or adjustments would have to be done in the project to ensure that fruehstArt can be integrated into the care of children with	In your opinion, what changes or adjustments to the project are needed so that it can be effectively integrated into the care of children with overweight and obesity in the long term? (T2)<br><br>Are there any components that should not be changed?<br>- If so, which should not be changed? (T2)<br>To what extent do you think that the two new offers of family outreach coaching and/or outpatient rehab should be components of care for children	What do you think should be improved at fruehstArt? (T1)<br><br>What changes to the project do you think would be necessary (so that you can support your child in implementing healthier lifestyle habits long-term)?<br>- Are there any aspects that should not be changed, and if so, which ones? (T2)

		overweight and obesity in the long term?<br>- Are there any components that should not be changed? If so, what should not be changed? (T2)<br><br>What specific adjustments need to be done to the structure/content of the coaching and the role of the coach within the project? (T2)<br><br>How do you think that the new offer of outpatient rehab in particular should be a component of care for children with overweight/obesity in the future? (T2)	with overweight/obesity in the future? (T2)	
	**Complexity**<br>(Perceived difficulty of implementation, reflected in duration, scope, implications, disruption, importance, complexity and number of steps required for implementation)	How realistic do you think the long-term implementation of fruehstArt is?<br>- Why? Why not? (T1 & T2)<br><br>Are there any conflicts that could affect the implementation of the programme?<br>- Which are these? (T1 & T2)	How realistic do you think the long-term implementation of fruehstArt is and why? (T2)<br><br>What obstacles have you encountered so far in implementing fruehstArt?<br>- Did the realisation of the project take more or less time and effort than you had imagined? (T1)	What do you think about the intensity of fruehstArt? (number of visits to the paediatrician, number of coaching sessions, etc.) (T2)<br><br>Which obstacles are there to achieving tasks and goals? Examples of why: personal motivation, frustration tolerance, lack of social support from family and friends, unfavourable living

		What helps you most in the realisation of your work as a coach?<br>What do you find particularly easy? What do you succeed at particularly well?<br>- App, study coordinators, materials, staff, etc. (T1 & T2)<br><br>In your opinion, what are the factors that promote the successful execution of tasks?<br>- Family Support etc. (T1)<br><br>How did you deal with the challenges you mentioned in our last interview?<br>- (Factors such as time, resources, experience, communication with paediatricians, families, etc.)<br>Were you able to break down barriers for yourself? If so, how? If not, why? (T2)<br><br>Have any problems or challenges arisen in the past few months that did not occur in the first few months of the project?<br>- New technical problems, staff etc.	What factors have helped you implement fruehstArt in your practice?<br>- Help from the study coordinators? Website, BMI calculator? (T1 & T2)<br><br>How did you deal with the challenges you mentioned in our last interview? (Case-specific list of the barriers mentioned in relation to your own day-to-day work)<br>- Factors such as time, resources, experience, communication with coaches, families, etc. Were you able to solve problems or break down barriers for yourself? If so, how? If not, why not? (T2)<br><br>Have any problems or challenges arisen in the last few months that did not occur in the first few months of the project?<br>- New technical problems, changes in the staff, etc.<br>If there were other problems in families: Do you have any case examples? (T2)<br><br>Have simplifying conditions been added in the last few months/have solutions been	environment, lack of time, combination with work, childcare/daycare etc. (T1 & T2)<br><br>How do you deal with these obstacles? What would help you? (T1)<br><br>How did you deal with these obstacles? What would help you or what has helped you? (T2)<br><br>To what extent do you believe that you will still be able to overcome some of these obstacles after the end of the project? (T1 & T2)<br><br>If rehab: Briefly describe the course of your rehab,<br>What barriers did you face as a family in participating in the rehab as planned and meeting the requirements? What did you find particularly helpful about the rehab and what less helpful?<br>What changes do you think could be made to enhance the benefits for families? (T2)

		If there were other problems in families: Do you have any case examples? (T2)<br><br>What brings/has brought you to your limits in the coaching sessions?<br>- How did you deal with these experiences? What helped you to master/ manage these challenges? (T2)<br><br>How difficult is it for you to agree on goals and tasks with the families? (T1)	found for the challenges you mentioned earlier? (T2)<br><br>What potential problems or complications do you foresee arising in the future? (T1)<br><br>What barriers do you see for other paediatricians to participate in a programme like fruehstArt? (T1 & T2)<br><br>To what extent did seasonal factors influence the process of fruehstArt (flu season etc.)? (T2)	
	**Design Quality and Packaging** *(Perceived quality in the way the intervention is bundled, presented and assembled)*	How have you experienced working with the fruehstArt app since we last spoke (functionality)?<br>- Were there any technical difficulties that you or the families experienced? If yes, please describe. (T1 & T2)<br><br>How has the fruehstArt app supported you in your work with families?<br>- If it has not, how and why not? (T1 & T2)<br>Do you consider the app (with a connection to an e-	Which materials did you use for the implementation of fruehstArt?<br>- How would you describe the quality of the materials? (T1 & T2)<br><br>In your opinion, how well did the fruehstArt materials (flyers, website, etc.) support the implementation process?<br>- What other materials would have been helpful to support the implementation of fruehstArt? (T1 & T2)	How do you experience using the fruehstArt app? How well did you manage using the fruehstArt app?<br>- Design, comprehensibility, usefulness... (T1 & T2)<br><br>Does it help you?<br>- If so, how and if not, why not?<br>How should the fruehstArt app be designed so that you use it and perceive it as a support in everyday life? (T2)<br><br>What do you think of the materials you received from your paediatrician and the coach?

		health platform for paediatricians) to be a (future) necessary component of the project? (T2)	How do you experience working with the e-health platform (functionality etc.)?<br>- How do you perceive the handling/design/technical problems? (T1 & T2)<br><br>To what extent has the e-health platform supported you in your work with families or did you perceive the application as additional work?<br>- Have you received any feedback on this from your healthcare professionals? (T1)<br><br>To what extent do you consider the BMI-SDS calculator from fruehstArt (for inclusion in the study) to be useful?<br>- Do you think that it will help you notice more children with a risk of obesity than the tools you have been using so far? (T1)	- Food pyramid, flyer etc. Would you like to receive more? (T1 & T2)<br><br>Would you like to continue using the fruehstArt app?<br>- Under what conditions? (T2)
**2. Outer Setting**	**Patient Needs and Resources**<br>*(The extent to which the needs of patients as well as the barriers and facilitators to meeting those needs are accurately known and prioritised by the organisation)*	In your opinion, what obstacles do the families face in realising the agreed tasks?<br>- In terms of time, finances, etc. (T1)<br><br>In your opinion, what obstacles are there for families to take part in the	In your opinion, how well does fruehstArt meet the families' needs?<br>- e.g. adaptation options to individual family situation/ individual needs of the child (T1 & T2)	Did the paediatrician respond to your needs and concerns?<br>- If not, why not? Please explain. (T1 & T2)<br><br>Do you have enough time with your paediatrician?<br>- Would you like more or less contact/time with the

		coaching sessions (e.g. time, communication, financial)?<br>- Do you have any specific case examples of families in this regard? (T1)<br><br>In your opinion, what obstacles do families face regarding the coaching sessions (e.g. time, communication, financial, cultural, parents' level of education)?<br>- Obstacles to participating in coaching sessions, but also to actively contributing to the change process. Do you have any specific case examples of families in this regard? (T2)<br><br>Can you identify any aspects that some families didn't like about the coaching?<br>- Were there similarities (in the change) in the process across many families? E.g. opening with time etc. (T2)<br><br>And what are the success factors in your opinion? (T2)<br><br>In your opinion, how well does fruehstArt meet the	To what extent did the families share their experiences from the coaching sessions with you?<br>- Describe the feedback. How did you deal with it? (T2)	paediatrician within the project? (T1 & T2)<br><br>How does the coach react to your needs?<br>- Individual family situation, traditions, financial resources, etc. (T1 & T2)<br><br>Is the content of the coaching adjusted to your family's needs? Can you give examples?<br>- Exercise, nutrition, sleep, media consumption. Would you like more content? (T1 & T2)<br><br>Where do you find it difficult to implement changes? Why? (T1 & T2)<br><br>Was there anything that prevented you from attending the paediatrician appointments (every 3 months)?<br>- Deadline pressure, work, illness, stress... (T2)<br><br>To what extent did you find the check-up appointments helpful? (T2)<br><br>Imagine other families who are in a similar situation to yours. What

		needs and possibilities of the families?<br>- Can you meet the parents' needs in the coaching sessions? (T1 & T2)<br><br>How did you experience the families' interaction with the fruehstArt app?<br>- Technical barriers, non-use, etc. If little/not at all used by families: What do you think would improve the use of the fruehstArt app for parents? (T1)<br><br>How have you experienced the cooperation with the families since our last conversation? Please describe. (T2)		could prevent these families from participating in this project?<br>- Lack of motivation from family members, financial hurdles (buying healthy food, sports club, etc.), time challenges<br>Where would you make time-related changes? (T1 & T2)
	**Cosmopolitanism**<br>*(The degree to which an organisation is networked with other external organisations)*	How did you find local support services for the families? (Existing networks via obesity centre? Previous employment?)<br>- To what extent has networking with local support services been successful/ to what extent do you believe that networking will be successful?	What is your opinion on the current form of cooperation and collaboration with the coaches? (T1 & T2)<br><br>How often did you coordinate/exchange with the coaches?<br>What content/topics played a particularly important role during the consultations/ exchanges?	Has your coach helped you to find offers in your local area that you can use with your child during or after the fruehstArt period?<br>- If so, which ones? To what extent would you have liked more or less support? (T1 & T2)<br><br>Have you already tried/used the suggested offers? (T1 & T2)<br>- If so, how were your first experiences? Were these offers

		What (potential) barriers were/are there for you as a coach?<br>What barriers do/did the families face in accessing these?<br>What factors facilitate their use? (T1)<br><br>How well did you manage to connect the families with the relevant support services?<br>- What barriers were there for the families to make use of them? (T2)<br><br>How have you identified local support services for the families since our last conversation? (Existing networks via obesity centre? Previous employment?)<br>- What barriers did you face as a coach in finding suitable offers? (T2)	Are/Were there any differences of opinion regarding the further course of the children's treatment? (T1 & T2)<br><br>How do you rate the cooperation with the study coordinators? (T1 & T2)<br><br>To what extent have you discussed fruehstArt with other paediatricians? (T1 &T2)<br><br>Would you recommend participation in the fruehstArt project to other paediatricians?<br>- If so, why? If not, why not? What would need to be changed so that you would recommend fruehstArt to others? (T1 &T2)	helpful to you? Please briefly describe why or why not.<br>If not, what were the reasons for not making use of them yet? (T1)<br>- Were these offers helpful to you? Please briefly describe why or why not. How were your first experiences? If not, what reasons did you have for not taking advantage of them? (T2)<br><br>Would you recommend fruehstArt to other families in a similar situation to yours? Why or why not? (T2)<br><br>Has your child participated in a rehabilitation programme within fruehstArt?<br>- Inpatient or outpatient? (T2)
	**Peer Pressure**<br>*(imitative or competitive pressure to implement a measure; usually because most or other major peer or competing organisations have already implemented a measure or to gain a competitive advantage)*			Did you feel free in your decision to participate in the intervention? (T1)<br><br>Do you know other families who participate/d in the fruehstArt project? If so, did this influence your decision to participate? (T1)

	**External Policies and Incentives** *(A broad construct that includes external strategies for disseminating interventions, including policies and regulations (government or other centralised bodies), external targets, recommendations and guidelines, performance-related pay, collaborations and public or comparative reporting)*		To what extent did you feel external influences or pressures (e.g. politics, measures, COVID-19) that may have influenced your decision to participate at fruehstArt?<br>- Rising obesity rates among children in your practice? (T1)	
**3. Inner Setting**	**Structural Characteristics** *(The social architecture, age, maturity and size of an organisation)*		What structural changes would be necessary in your practice to establish fruehstArt long-term? (Staff structure, time schedules, networking with other stakeholders, finances, further training programmes, etc.) (T2)	
	**Networks and Communications** *(The type and quality of social networks and the type and quality of formal and informal communication within an organisation)*	How did you experience communication within the fruehstArt project (with paediatricians, study coordinators, parents, IGKE, rehab)?<br>- Would you have preferred a different style of communication? Where and how? (T1 & T2)<br><br>Have there been any changes since we last spoke? (T2)	How did you experience communication within the fruehstArt project (study coordinators, coaches, IGKE)?<br>- Were there any changes you would have requested?<br>Were there any conflicts that could have affected the implementation of the project? Which ones? (T1 & T2)<br><br>To what extent do you feel sufficiently supported? (T1 & T2)	How do you generally perceive the communication with the paediatrician in the project?<br>- Are there or have there been communication difficulties?<br>If so, what kind of communication would you prefer? (T1 & T2)<br><br>How do you feel about the communication with the coach during the coaching session?<br>- Are there any communication difficulties? If so, what kind of communication would you prefer? Language barriers? Did

		How did you experience the regular Jour Fixes?<br>- Were your concerns adequately discussed here? Were open questions clarified? Did you have sufficient opportunity to exchange with the other coaches? (T1)<br><br>Who do you contact if you have questions about fruehstArt or its implementation and how?<br>- How available are these people? (T1 & T2)	Who do you contact if you have questions about fruehstArt or its implementation and how?<br>- How available are these people? (T1 & T2)	the coach use simple/understandable language? (T1 & T2)<br><br>How did communication with the coach work outside the coaching sessions (e.g. via the fruehstArt app, phone calls)? (T1 & T2)<br><br>If so, who are your main contacts in case of problems? To what extent do you feel sufficiently supported?<br>- paediatrician, coach, study coordinators? (T1 & T2)<br><br>How was the communication with the coach during the rehab phase?<br>- Were there any contradictions between what you learnt in rehab and what the coach advised? (T2)<br><br>If person XY is responsible for it/ or doesn't care. How does this affect communication within the family?<br>- Are there differences of opinion, disputes (also regarding the realisation of tasks and objectives...)? (T1 & T2)
	**Implementation Climate**		How willing do you think the staff at your practice are to	What do you think: Under what conditions would families in a

	(The ability to change, the shared willingness of the people involved to engage with an intervention and the extent to which the use of this intervention is rewarded, supported and expected within their organisation)		implement the fruehstArt project as planned?<br>- Why? Can you explain? (T1)<br><br>How willing were the staff at your practice to implement the fruehstArt project as planned?<br>- In your opinion, was there too little capacity or too little willingness at one point? (T2)<br><br>To what extent have you noticed changes in the implementation of fruehstArt in your practice?<br>- In relation to your own working methods, the practice staff, etc. (T2)	similar situation to yours participate in this project? (T2)
	**Tension for Change**<br>*(The extent to which stakeholders perceive the current situation as intolerable or in need of change)*		In your opinion, how important is a project like fruehstArt for improving the care situation of families with children who are overweight/obese? (T1 & T2)<br>- Do many patients in your practice have a need for a project like fruehstArt? (T1)	
	**Compatibility**<br>*(The degree of concrete alignment between the meaning and values that the people involved associate with the intervention, how these align with their own norms, values and perceived risks and needs,*		How well could fruehstArt be integrated into the existing workflows in your practice?<br>- To what extent could fruehstArt replace or change existing processes/ procedures? (T1 & T2)	How easy or difficult is it for you to attend the coaching sessions as scheduled?<br>- Time pressure, combination with work, childcare, etc. (T1 & T2)

	and how the intervention fits into existing workflows and systems)		To what extent did fruehstArt interfere with existing processes/procedures? (T2)	To what extent have you noticed differences in attitudes and cultural views between you and the coach? Did these influence the coaching process?<br>- Different ideas about cooking, eating and sports behaviour etc. Do the suggested recipes match the family's taste? (T1 & T2)<br><br>How well did the rehab fit into the family's daily routine? (T2)
	**Relative Priority**<br>*(Shared perception of the importance of implementation within the organisation by the individual)*		What priority does the implementation of fruehstArt have compared to the existing treatment processes in your practice (or to other programmes running at the same time)?<br>- Does the implementation of fruehstArt create any conflicts with other aspects or activities of your work? (T1 & T2)	
	**Organizational Incentives and Rewards**<br>(Extrinsic incentives such as target agreement bonuses, performance appraisals, promotions and salary raise as well as less tangible incentives such as a higher reputation or respect)		How do you rate the remuneration for your work within the project? (T1)	What forms of support could you imagine?<br>- Financial support, additional support from the paediatrician, coach etc. (T1 & T2)<br><br>Would you also consider the project for your family if you had to pay for it? (T2)
	**Goals and Feedback**	To what extent do you receive feedback on your	Do you receive feedback from the fruehstArt team/study	There may have already been instances where you were unable

	(The extent to which goals are clearly communicated, implemented and reported back to employees, and the alignment of this feedback with the goals)	coaching work from the fruehstArt team/ paediatricians?<br>- If so, in what form do you receive it?<br>If so, is the feedback helpful?<br>If not, would you have preferred (more/different) feedback? (T1)<br><br>To what extent are you aware of the overarching project goals you should achieve with the families during the intervention period?<br>- To what extent do you miss further agreements (with the IGKE, the paediatricians, the AG coaches) regarding the goals you want to achieve? - BMI-SDS reduction, quality of life, health literacy, etc. (T1)	coordinators on your work within fruehstArt?<br>- If so, in what form did you receive it? If so, was the feedback helpful?<br>If not, would you have liked (more) feedback / Were you available for this feedback? (T1 & T2)<br><br>To what extent are there reactions to the requirements of the project? Or other feedback (praise or complaints)?<br>- In case of complaints: Were there any conflicts? How do you deal with them? Do you have any specific examples of families in this regard? (T1)<br><br>To what extent were there reactions to the requirements of the project? Or other feedback (praise or complaints)?<br>- Were there any conflicts? (T2)	to achieve the defined goals and tasks. How did your coach proceed then? Did you adjust the goals?<br>- Change in daily and weekly planning? (T1 & T2)
	**Learning Climate**<br>(A climate in which a) leaders express their own fallibility and their need for support and input from team members; b) team members feel that they are important, valued and knowledgeable partners in the	If you notice a problem for yourself or the families within fruehstArt, what actions do you take?<br>- To what extent do you feel that your concerns are acknowledged and addressed?	If you notice a problem for yourself or the families within fruehstArt, what actions do you take?<br>- Is your feedback taken seriously? (T1 & T2)	To what extent do you feel that your coach listens to your problems and concerns?<br>- Do you feel comfortable talking openly with your coach about private problems that are barriers to achieving your goals?

	change process; c) individuals feel mentally safe to try new methods; and d) there is sufficient time and space for reflective thinking and evaluation)	Are your feedback and concerns taken seriously? (T1 & T2)<br><br>How well have you been coping with this type of self-organisation so far? (T1)<br><br>How has your work as a coach changed since our last interview?<br>- Have you noticed any changes in your own working method? (e.g. through increased experience)? If so, please describe. (T2)		(Stress at work, financial difficulties, problems with the education of your child, etc.) Are your concerns taken seriously? (T1 & T2)<br>Have there been any problems within the project so far? If so, which ones?<br>- Problems with the coach, the app, the paediatrician etc. (T1 & T2)
	**Available Resources**<br>*(The number of resources provided for implementation and ongoing operations, including money, training, education, space and time)*	In your opinion, did you have enough time with the families? (to optimally realise the goals of the project?)<br>- If not, how much time would have been needed (for exercise, education, nutrition, sleep, media consumption)?<br>And 'what time': more preparation and follow-up time, more time per home visit or more home visits in total? (T2)	Did you feel sufficiently prepared to implement the fruehstArt project? (T1)<br><br>Are there any other resources you would like to have received? (T1)<br><br>To what extent do you think you had enough time with the families?<br>- If not enough, how much additional time would you have needed? And for which part/conversation content? (T2)	

		Did the training adequately prepare you for working with the families?<br>- If so, why? If not, why not? What, if anything, did you lack to feel well prepared? (T1)<br><br>How did you perceive the refresher training? (T1)	Do you consider the e-health platform to be a (future) necessary component of the project? If so, why? If not, why not? (T2)<br><br>Have you participated in a training course on motivational interviewing?<br>- If so, which one and why? If not, why not?<br>If so, how did you perceive the training? Was any content missing? (T1)<br><br>Did you have sufficient resources (monetary/time) for the realisation of fruehstArt? (T1)	
	**Access to Knowledge and Information**<br>*(Easy access to understandable information and knowledge about the intervention and its integration into workflows)*	Did you feel prepared by the training to fulfil the tasks and responsibilities assigned to you?<br>- All e-learning and face-to-face training and follow-up appointments. Can you explain this? (T1)<br><br>Did you receive sufficient information and materials during the training that you can/ could use in the coaching sessions?	Did the materials you received in advance help you to understand the fruehstArt project?<br>- If not, why not? What else would you have liked/needed? Were you able to clarify any questions/problems the families had with the help of these materials? (T1)	How do you like the supporting information provided in the fruehstArt app?<br>- Tips & tricks, recipes etc. (T1 & T2)

		(e.g. psychosocial medical history form, food pyramid, handouts, recipe database, plate rule).<br>- If so, what is particularly helpful? Have you been able to resolve any questions/problems of the families?<br>If not, why not? (T1)<br><br>Which materials provided by the project have you been able to use most effectively in your work with the families? (food pyramid, handouts, recipe database, plate rule)<br>- Were you able to resolve any questions/problems?<br>Would you have preferred more or different materials? (T2)		
**4. Characteristics of Individuals**	**Knowledge & Beliefs about the Intervention**<br>*(The individual's attitude towards the intervention and the value they place on it, as well as knowledge of facts, findings and principles related to the intervention)*	To what extent do you think fruehstArt will be successful (reduction of BMI-SDS, quality of life, etc.)?<br>- What (potential) impact do you think fruehstArt has on parents and children? (T1)<br><br>Do you think that fruehstArt was effective (reduction of	Do you believe that the fruehstArt project will be effective (reduction of BMI-SDS, quality of life, etc.)? (T1)<br><br>To what extent do you think fruehstArt was effective (reduction of BMI-SDS, quality of life, etc.)?	

		BMI-SDS, quality of life, etc.)? In your opinion, did anything change in this respect during the project?<br>- What influence do you think fruehstArt has on parents and children? What changes have you experienced in the families? (T2)	- Can you give us some examples? (T2)<br><br>How well do you think fruehstArt will be accepted by other paediatricians in the future? (T1 &T2)<br><br>How do you rate the course of the project? Would you say that fruehstArt was fully/well accepted by families? (T2)	
	**Self-efficacy**<br>*(The individual believes in their own ability to carry out measures to achieve the implementation goals)*			How confident do you feel about continuing to implement the contents of fruehstArt and to set new goals yourself even after the project has ended? (T2)<br><br>Why are you (so) confident (or not so confident)? What would have to happen to increase your confidence? (T2)
	**Individual Stage of Change**<br>*(Description of the stage a person is at in their progress towards skilled, enthusiastic and sustained use of the intervention)*			When you think about your time in the project so far. Have you already been able to implement concrete changes in everyday family life? What are these? If not, why not?<br>- What have you achieved/changed as a family/ or your child during the project period so far? (T1 & T2)

	**Individual Identification with Organization**<br>*(A broad construct that refers to how the individual perceives and relates to the organisation and to what extent they feel committed to it)*	How much influence do you have on the organisation and implementation of the coaching sessions? (T1 & T2)<br><br>How much own room for manoeuvre do you feel you have (e.g. in trying out new things) etc.? How do you rate this? (T1 & T2)<br>Have you been able to improve work processes with it? (T2)<br><br>What are the biggest challenges in your position/work? (T1)<br><br>What do you rate as positive about your work at fruehstArt? (T1 & T2)<br>- Perception of changes compared to the first interview? (T2)<br><br>What do you rate as negative about your work at fruehstArt? (T1 & T2)	What are the biggest challenges for you within fruehstArt? (T1 & T2)<br><br>What do you rate as positive about your involvement in fruehstArt? (T1 & T2)<br>- Perception of changes compared to the first interview? (T2)<br><br>What do you rate as negative about your involvement? (T1 & T2)	Which aspects of fruehstArt did you and your child like best and which did you like least? (T2)
	**Other Personal Attributes**<br>*(A broad construct that also includes other personal characteristics such as tolerance of ambiguity, intellectual ability,*	To what extent has the motivation and commitment of the families changed throughout the duration of the project? (T2)	Has your participation in fruehstArt changed the way you communicate and interact with your patients/families? (T1)	

	motivation, values, competence, capacity and learning style)		How do you perceive the motivation and willingness of parents to collaborate on fruehstArt? (T1)<br><br>To what extent has the motivation and commitment of the families changed over the duration of the project?<br>- Do you have a case example? Were there similarities in the process across many families? (T2)	
**5. Process**	**Planning**<br>*(The extent to which a plan or method of behaviour and tasks for implementing an intervention are developed in advance, and the quality of these plans or methods)*	How did you find the introduction to the fruehstArt app?<br>- Were all questions and uncertainties satisfactorily clarified? What would you suggest to improve the introduction to the app? (T1)	Please describe how fruehstArt was introduced in your practice?<br>- Who was involved in the planning process? Examples: Integration into practice management, accounting, documentation, etc. (T1)<br><br>How did you perceive the introduction to the e-health platform?<br>- Could all questions and uncertainties be clarified? What would you improve? (T1)	How did the enrolment in the project with the paediatrician work? (T1)<br><br>How did you perceive the first few weeks of coaching?<br>- How did you feel during the first few weeks of the coaching? (T1)<br><br>How did you perceive the initial meeting? (T1)<br><br>How was the first contact with the coach?<br>- Initial telephone contact (T1)<br><br>How was the first meeting?<br>- How did you like it? How did the first meeting proceed? (T1)

	**Opinion Leaders** *(Individuals in an organisation who formally or informally influence the attitudes and beliefs of their colleagues in relation to the implementation of the intervention)*			Who in your family participates most frequently in the coaching sessions? Who is most involved?<br>- Mother, father, grandmother, babysitter... (T1 & T2)<br><br>Is there a specific division of tasks for executing the tasks and goals that are defined in the coaching sessions?<br>- Who does the shopping? Who cooks? Who does the sports activities? (T1 & T2)
	**Important components** *(Diverse strategies to attract and involve key stakeholders in the implementation or utilisation of the innovation (e.g. through social marketing, education, role modelling, training)*		-How would you describe the motivational interviewing with the families? (T1 & T2)<br><br>- If you attended the training: Were you able to apply the techniques you were taught? Did you have enough time to do so? If not, how much time would you have needed? (T1)	How did you find out about the project?<br>- Did your paediatrician approach you about your child's weight or were you seeking a dialogue? (T1)
	Patient/Consumer *(Diverse strategies for attracting and involving patients/consumers in the implementation or use of the innovation (e.g. through social marketing, education, role modelling, training)*	To what extent did you observe cultural differences between the families? (Eating behaviour, activity behaviour, routines etc.)<br>- Influence on cooking, views on sports behaviour, etc. (T2)	What were the reasons why some families did not want to participate in the fruehstArt project? (T1)<br><br>What were the reasons why families dropped out during the project?<br>- e.g. excessive demands etc. (T2)	Why did you decide to participate in the project? What expectations did you have regarding your participation in fruehstArt?<br>- Have you set yourself a specific goal? (T1)<br><br>Have your expectations of the project been fulfilled so far?

		Did you feel accepted by the families in your role as a coach?<br>- Were there any influences of age, gender, culture?<br>Can you give examples? (T2)	How exactly does the recruitment of families work? - What challenges do you encounter when recruiting?<br>What are favourable factors from your point of view?<br>What steps are you taking to encourage parents to make use of the programme? (T1)<br><br>How do you generally feel about raising the issue of overweight/obesity with parents?<br>- To what extent did parents seek a dialogue with you themselves?<br>Did the training in motivational interviewing help? (T1)	- If so, why? If not, why not? (T1 & T2)<br><br>How do you evaluate the joint definition of goals and tasks with the coach? (T1 & T2)<br>- What is going particularly well and what less so? (T1 & T2)<br><br>When you attempt to implement the goals and tasks in your daily life, do you find it enjoyable, or do you find it annoying?<br>- Are there tasks that you enjoy more or less than others? On which topics: exercise, nutrition, sleep, media consumption? Please explain. (T1 & T2)<br><br>What are your wishes for the further course of the project?<br>- For yourselves as a family? For your child? (T1)<br><br>What has helped you to achieve goals and tasks?<br>- Family support, tips from the coach? (T1)<br><br>To what extent have you already received support from your coach on how to help your child be more active/ eat healthier/

				reduce media time/ sleep more and better?<br>- Give a few examples... (T1 & T2)
	**Executing/Fidelity**<br>*(realisation or completion of the implementation according to plan)*	To what extent have you been able to conduct the home visits as planned (based on the document 'Procedure for home visits')?<br>- What could not be executed as planned? Why not? (T1 & T2)<br><br>Have you changed anything in your coaching approach since our last interview? (own motivation, techniques, expectations, attitude etc.?) (T2)	Was there anything that prevented you from starting the implementation of fruehstArt as planned?<br>- If so, what and how did you handle it? (T1)<br><br>Were you able to implement/organise the regular counselling appointments for the families as planned?<br>- Please explain. (T2)	

	**Reflecting and Evaluating** *(Quantitative and qualitative feedback about the progress and quality of implementation, accompanied by regular personal and joint meetings about progress and experiences)*	Were there any effects within the families that you did not expect? (e.g. weight gain, radical lifestyle changes, etc.)<br>- If so, what were the reasons for this? (T2)<br><br>Have any families you looked after left the project?<br>- If so, what were the reasons for this? (T2)<br><br>To what extent have cultural differences between you and the families impacted the coaching process? (T2)	Were there any effects within the families that you did not expect? In your opinion, what are the most significant effects that you can/could recognise?<br>- e.g. weight gain, radical lifestyle, etc. (T2)<br><br>What were the reasons why families decided not to participate in fruehstArt? What percentage (roughly estimated) decided for/against it after the motivational counselling?<br>- What were their reasons for doing so?<br>How did the control group families react to the fact that they did not receive coaching sessions? Did this lead to drop-outs in the control families? (T2)<br><br>How have you experienced the cooperation with the families so far? Please describe.<br>- This refers to the cooperation from enrolment to the current status of the family. How do the quarterly appointments with the families work? (T1 & T2)	
